# Independent Origin and Global Distribution of Distinct *Plasmodium vivax* Duffy Binding Protein Gene Duplications

**DOI:** 10.1371/journal.pntd.0005091

**Published:** 2016-10-31

**Authors:** Jessica B. Hostetler, Eugenia Lo, Usheer Kanjee, Chanaki Amaratunga, Seila Suon, Sokunthea Sreng, Sivanna Mao, Delenasaw Yewhalaw, Anjali Mascarenhas, Dominic P. Kwiatkowski, Marcelo U. Ferreira, Pradipsinh K. Rathod, Guiyun Yan, Rick M. Fairhurst, Manoj T. Duraisingh, Julian C. Rayner

**Affiliations:** 1 Malaria Programme, Wellcome Trust Sanger Institute, Hinxton, United Kingdom; 2 Laboratory of Malaria and Vector Research, National Institute of Allergy and Infectious Diseases, National Institutes of Health, Rockville, Maryland, United States of America; 3 Program in Public Health, University of California, Irvine, California, United States of America; 4 Department of Immunology and Infectious Diseases, Harvard T.H. Chan School of Public Health, Boston, Massachusetts, United States of America; 5 National Center for Parasitology, Entomology, and Malaria Control, Phnom Penh, Cambodia; 6 Sampov Meas Referral Hospital, Pursat, Cambodia; 7 Department of Medical Laboratory Sciences and Pathology, College of Public Health and Medical Sciences, Jimma University, Jimma, Ethiopia; 8 Department of Chemistry, University of Washington, Seattle, Washington, United States of America; 9 MESA-ICEMR, Goa Medical College and Hospital, Bambolim, Goa, India; 10 Department of Parasitology, Institute of Biomedical Sciences, University of São Paulo, Brazil; Walter and Eliza Hall Institute, AUSTRALIA

## Abstract

**Background:**

*Plasmodium vivax* causes the majority of malaria episodes outside Africa, but remains a relatively understudied pathogen. The pathology of *P*. *vivax* infection depends critically on the parasite’s ability to recognize and invade human erythrocytes. This invasion process involves an interaction between *P*. *vivax* Duffy Binding Protein (PvDBP) in merozoites and the Duffy antigen receptor for chemokines (DARC) on the erythrocyte surface. Whole-genome sequencing of clinical isolates recently established that some *P*. *vivax* genomes contain two copies of the *PvDBP* gene. The frequency of this duplication is particularly high in Madagascar, where there is also evidence for *P*. *vivax* infection in DARC-negative individuals. The functional significance and global prevalence of this duplication, and whether there are other copy number variations at the *PvDBP* locus, is unknown.

**Methodology/Principal Findings:**

Using whole-genome sequencing and PCR to study the *PvDBP* locus in *P*. *vivax* clinical isolates, we found that *PvDBP* duplication is widespread in Cambodia. The boundaries of the Cambodian *PvDBP* duplication differ from those previously identified in Madagascar, meaning that current molecular assays were unable to detect it. The Cambodian *PvDBP* duplication did not associate with parasite density or *DARC* genotype, and ranged in prevalence from 20% to 38% over four annual transmission seasons in Cambodia. This duplication was also present in *P*. *vivax* isolates from Brazil and Ethiopia, but not India.

**Conclusions/Significance:**

*PvDBP* duplications are much more widespread and complex than previously thought, and at least two distinct duplications are circulating globally. The same duplication boundaries were identified in parasites from three continents, and were found at high prevalence in human populations where DARC-negativity is essentially absent. It is therefore unlikely that *PvDBP* duplication is associated with infection of DARC-negative individuals, but functional tests will be required to confirm this hypothesis.

## Author Summary

Malaria parasites must be adaptable to evade the human immune system and successfully transmit themselves to different individuals. Key to this adaptability is the fact that malaria parasite genomes are highly variable, containing mutations ranging from simple small changes in DNA sequence to complex large-scale changes in the number of copies of individual genes. Some samples of *Plasmodium vivax*, the parasite that causes most malaria episodes outside Africa, have recently been found to have duplicated the gene encoding Duffy Binding Protein, which enables *P*. *vivax* parasites to recognize and invade human red blood cells. By studying parasites from Cambodian patients with *P*. *vivax* malaria, we have discovered that there are actually two different types of gene duplication, with the new duplication type identified in this study present in 20% to 38% of Cambodian *P*. *vivax* isolates tested. The same gene duplication was also found in parasites from Brazilian and Ethiopian patients with *P*. *vivax* malaria, suggesting that variation in this gene is more complex and common than previously thought. The functional significance and origin of these two gene duplications requires further study.

## Introduction

*Plasmodium* parasites must recognize and invade erythrocytes to multiply and cause clinical disease in humans. Erythrocyte invasion is a complex multi-step process involving multiple protein-protein interactions between the extracellular *Plasmodium* merozoite and its target erythrocyte. While *P*. *falciparum* merozoite proteins have some overlapping and partially redundant interactions with several different erythrocyte receptors [1], *P*. *vivax* merozoites appear to rely heavily on the interaction between *P*. *vivax* Duffy Binding Protein (PvDBP) and the erythrocyte Duffy antigen receptor for chemokines (DARC) to invade human erythrocytes [2–4]. There are numerous common genetic variants at the *DARC* locus, including the DARC-negative or FY*B^*ES*^/*B^*ES*^ genotype, where a polymorphism in the *DARC* promoter eliminates expression of DARC specifically in erythroid cells, producing an Fy(a-b-) phenotype [5]. DARC-negativity is nearly ubiquitous in West and Central Africa and, given that erythrocyte invasion is essential for parasite survival, is associated with protection against *P*. *vivax* infection [6]. PvDBP is therefore a high-priority *P*. *vivax* vaccine candidate [7]. The essentiality of the PvDBP-DARC interaction for *P*. *vivax* invasion has recently been challenged by the discovery of confirmed *P*. *vivax* infections in DARC-negative individuals in Madagascar [8], a finding that is consistent with several independent reports of *P*. *vivax* infections in DARC-negative individuals in West and Central Africa [9]. The molecular basis of *P*. *vivax* invasion into DARC-negative erythrocytes is currently unknown, although it may still involve PvDBP.

Given this focus on PvDBP for both vaccine and biological studies, understanding the extent and functional consequences of *PvDBP* variation is important. Previous attention to *PvDBP* variation has focused on single-nucleotide polymorphisms (SNPs), which are common and appear to result in strain-specific immune responses [10,11]. Recent whole-genome sequencing of a *P*. *vivax* strain from Madagascar identified another source of variation, namely duplication of the entire *PvDBP* locus [12]. A simple PCR assay based on these whole-genome sequence data revealed that *PvDBP* duplication was present in more than half of Malagasy *P*. *vivax* samples, and in much lower proportions of East African, Asian, and South American samples. This finding has since been confirmed by whole-genome sequencing of >200 *P*. *vivax* isolates from around the world, where evidence for duplication based on increased sequence coverage was found in 35% of Cambodian isolates [13].

The functional consequences of *PvDBP* duplication are unknown, although its high prevalence in Madagascar raised the intriguing possibility that it is somehow associated with successful *P*. *vivax* infection of DARC-negative individuals. Whether the *PvDBP* duplication arose only once and spread globally, or arose independently more than once, which would suggest localized selection pressure, is also unknown. While such locally-selected copy number variations (CNVs) in *Plasmodium* genes are known to associate with drug resistance, such as CNVs at the *PfMDR1* locus in association with mefloquine resistance [14,15], such CNV selection has not yet been associated with genes involved in fundamental biological processes such as erythrocyte invasion. To address the origins of CNVs at the *PvDBP* locus, we investigated the prevalence and nature of *PvDBP* duplications in 37 *P*. *vivax* samples collected in a clinical study of human genetic resistance to *P*. *vivax* malaria in Cambodia [16], which were subsequently sequenced by the Malaria Programme at the Wellcome Trust Sanger Institute (WTSI) as part of a larger study [13]. This identified a new type of duplication, which was then validated in >350 samples from all three continents where *P*. *vivax* infections are found.

## Methods

### Patient samples and ethics statement

Cambodia: During each annual malaria season (June-December) from 2008 to 2011, patients presenting with malaria symptoms were screened at Sampov Meas Referral Hospital in Pursat. A total of 898 patients were diagnosed with *P*. *vivax* malaria and participated in a study of human genetic resistance to this disease. The clinical protocol was approved by the National Ethics Committee for Health Research in Cambodia and the NIAID Institutional Review Board in the United States (ClinicalTrials.gov identifier NCT00663546). Following written informed consent, patients provided a venous blood sample, and DNA was extracted from 200 μl of whole blood using a QiaAmp kit (Qiagen, USA). Of these samples, 49–50 samples per year were randomly selected for analysis.

Ethiopia: During the peak malaria season (September-November) in 2014, patients presenting with malaria symptoms were screened at health centers in Jimma. Finger-prick blood samples (50 μl) were collected and screened for *P*. *vivax* infection by both microscopy and nested PCR. A total of 25 patients were diagnosed with *P*. *vivax* malaria and provided written informed consent to participate in this study. The clinical protocol was approved by the institutional review boards of Jimma University in Ethiopia and University of California, Irvine in the United States.

India: From July 2013 to December 2015, patients presenting with malaria symptoms were screened at Goa Medical College by both rapid diagnostic test (RDT) and microscopy. Patients with *P*. *vivax* monoinfection were then referred to the Malaria Evolution in South Asia (MESA) International Center for Excellence in Malaria Research (ICEMR) study team. Written informed consent was obtained from adult patients and the parents or guardians of minors; verbal assent was provided by children aged 8–17 years. Subjects provided 4–6 ml of venous blood, and parasite species was confirmed by RDT (FalciVax, Zephyr Biomedicals, India) and microscopy. A random selection of 72 *P*. *vivax* monoinfections (from a total of 838 *P*. *vivax* monoinfections diagnosed during the study period) was tested for the presence of *PvDBP* duplication. DNA was extracted from 50–500 μl of whole blood using a QiaAmp kit (Qiagen, USA). The clinical protocol was approved by the institutional review boards of Goa Medical College and Hospital, University of Washington, NIAID Division of Microbiology and Infectious Diseases, and Government of India Health Ministry Screening Committee.

Brazil: From 2008 to 2011, patients presenting with malaria symptoms at clinics in Acrelândia and Plácido de Castro [17] were screened by microscopy. Patients with *Plasmodium* infection provided 15-ml venous blood samples that were further examined for malaria parasites by a quantitative real-time PCR specific for the *18S rRNA* gene. Written informed consent was obtained from adult patients and the parents or guardians of minors. A random selection of six *P*. *vivax* monoinfections was tested for the presence of *PvDBP* duplication. These samples were collected under protocols approved by the Institutional Review Board of the Institute of Biomedical Sciences, University of São Paulo, Brazil (936/CEP, 2010).

Brazil: From June 2014 to September 2015, patients aged 5–63 years presenting with malaria symptoms at clinics in the urban area of Mâncio Lima were screened by microscopy. Patients positive for *P*. *vivax* infection were enrolled in a clinical trial of *P*. *vivax* response to chloroquine alone vs. chloroquine plus primaquine (ClinicalTrials.gov Identifier: NCT02691910) and provided 40-ml venous blood samples. Written informed consent was obtained from adult patients and the parents or guardians of minors. A random selection of 54 *P*. *vivax* monoinfections was tested for the presence of *PvDBP* duplication. These samples were collected under protocols approved by the Institutional Review Board of the Institute of Biomedical Sciences, University of São Paulo, Brazil (1169/CEP, 2014).

A random selection of samples were tested from Cambodia, Ethiopia, India and Brazil, with numbers based on sample availability.

### Identification of a novel *PvDBP* duplication in Cambodian *P*. *vivax* isolates

A subset of 37 Cambodian patients with *P*. *vivax* malaria provided an 8-ml blood sample, which was depleted of leukocytes using CF11 prior to DNA extraction [18]. These samples were sequenced at the WTSI using Illumina sequencing technology, as described [13]. As part of the genome analyses, the generated sequences were mapped individually to the *P*. *vivax* Sal1 reference genome (http://plasmodb.org/common/downloads/release-10.0/PvivaxSal1/fasta/data/PlasmoDB-10.0_PvivaxSal1_Genome.fasta) [19] using bwa version 0.5.9-r16 with default parameters [20]. In the present study, the resulting assembly bam files were reviewed in the region containing *PvDBP* (chromosome 6: 976329–980090, GenBank accession ID = PVX_110810) using the Artemis genome viewer [21] or Lookseq [22]. Mate pairs that were oriented tail-to-tail, and thus signified that *PvDBP* was duplicated, were visualized in either Artemis using a “non-proper pair” read filter or Lookseq using the “face-away” setting. To compare the breakpoints in Cambodian and Malagasy isolates, the read data for the Malagasy *P*. *vivax* M15 isolate (with a duplicated *PvDBP*) (SRA accession SRX266275) were mapped to the reference *P*. *vivax* Sal1 genome using bowtie2 v2.1.0 [23] with default parameters, except for counting overlapping reads as concordant pairs (--dovetail). In all sequences the *PvDBP* genes were reviewed for evidence of intra-isolate SNPs with a frequency of 0.3–0.7 and a minimum read coverage of 5 for both reference and alternative alleles using all SNPs called using GATK’s Haplotype caller with default settings [24,25]. Average coverage across all 14 reference *P*. *vivax* Sal1 chromosomes and the *PvDBP* locus (chromosome 6: 976329 to 980090) was computed using GATK’s DepthOfCoverage tool and compared to estimate *PvDBP* amplification [24,25].

### *PvDBP* duplication-specific PCR assay

We used previously-published primers and new primers to identify *PvDBP* duplications in our samples. Previously-published primers from Menard *et al*. [12] are as follows: primer pair 5’-CCATAAAAGGTAGGAAATTGGAAA-3’ (AF) and 5’-GCATTTTATGAAAACGGTGCT-3’ (AR), which amplifies a 613-bp region surrounding the Malagasy isolates’ breakpoint at position 982947, and primer pair 5’-TCATCGAGCATGTTCCTTTG-3’ (BF) and 5’-TTGCACGTACTCGAAACTCAG-3’ (BR), which amplifies a 643-bp region surrounding the breakpoint at position 974770. BF+AR are predicted to amplify a 612-bp product that contains the junction between the *PvDBP* copies in isolates with duplications matching those in Malagasy isolates (based on GenBank accession ID KF159580). BF+BR are predicted to amplify the identical breakpoint in Cambodian isolates. Primer pair 5’-ACGCGATGTATCTTCTTTTCA-3’ (AF2) and 5’-TAGAACGCACAGTTATTGGC-3’ (AR2) are designed to amplify a 657-bp region surrounding the breakpoint at position 982100 in Cambodian samples. AF2+AR2 are expected to amplify the region in samples with or without the *PvDBP* duplication and were used as positive controls. In samples with the Cambodian *PvDBP* duplication, BF+AR2 are expected to amplify a 736-bp product that contains the junction between the *PvDBP* copies. In Malagasy samples, BF+AR2 are expected to amplify a 1584-bp product containing the *PvDBP* duplication. These primers are opposite-facing in samples without the duplication and thus are not expected to produce a product. Duplication PCR products were capillary sequenced, and chromatograms were reviewed to determine the exact breakpoint. PCR reaction volumes contained 20 μl Platinum PCR Supermix (Thermo Fisher Scientific, USA), 1 μl DNA template, and 0.5 μl each primer (10 μM working stocks). PCR conditions were: 94°C for 2 min, followed by 35 cycles of 94°C for 20 s, 55°C for 30 s, and 68°C for 60 s, followed by a 4-min extension.

### Duffy genotype PCR assay

To genotype the Duffy blood group (Fy), we used the following published primers (see SI Appendix C in [8]): primer pair 5’-GTGGGGTAAGGCTTCCTGAT-3’ (Fy-F) and 5’-CAGAGCTGCGAGTGCTACCT-3’ (Fy-R), which amplifies a 1100-bp region spanning the GATA-1 transcription factor-binding site that determines DARC expression on erythrocytes [FY*B versus FY*B^*ES*^ in the promotor region of *PvDBP* (-33, TTA**T**CT for wild-type *versus* TTA**C**CT for erythrocyte silent FY*B^*ES*^/FY*B^*ES*^)] through the exon-two SNPs determining FY*A versus FY*B genotypes [codon 42, G**G**T (G) for FY*A versus G**A**T (D) for FY*B] and the FY*B^*weak*^ genotype (codon 89, **C**GC (R) for FY*B versus **T**GC (C) for FY*B^*weak*^). The FY*B^*weak*^ genotype is only known to be associated with the FY*B genotype. PCR reaction volumes contained 20 μl Platinum PCR Supermix, 1 μl DNA template, and 0.5 μl each primer (10 μM working stocks). PCR conditions were: 94°C for 2 min, followed by 35 cycles of 94°C for 20 s, 58°C for 30 s, and 68°C for 60 s, followed by a 4-min extension. PCR products were capillary sequenced using the following four published primers: 5’- GTGGGGTAAGGCTTCCTGAT-3’ (Fy-F), 5’- CAAACAGCAGGGGAAATGAG-3’ (GATA-1-R), 5’-CTTCCGGTGTAACTCTGATGG-3’ (Exon2-F), and 5’- CAGAGCTGCGAGTGCTACCT-3’ (Fy-R) (see SI Appendix C in [8]). Sequences were aligned to a reference sequence for *DARC* (GenBank accession number S76830.1, http://www.ncbi.nlm.nih.gov/nuccore/S76830.1), and chromatograms were visually inspected to determine *FY* genotypes. We determined the sequence at all three SNP sites in 129 samples, and at only the FY*A and FY*B sites in another 33 samples.

### *PvDBP* haplotypes and phylogeny

*PvDBP* haplotypes of the most abundant clone in each of 37 whole-genome-sequenced Cambodian samples and one Malagasy *P*. *vivax* M15 sample were reconstructed using GATK’s HaplotypeCaller v 3.4 [24,25], setting “sample ploidy” to 1 for the *PvDBP* locus (chromosome 6: 976329 to 980090). The resulting vcf file was used with GATK’s FastaAlternateReferenceMaker to construct FASTA files representing one *PvDBP* gene in each sample. The haplotype of the *PvDBP* region for *P*. *vivax* M15 was identical to the sequence deposited in GenBank (accession ID = KF159580), except for calling the majority “T” at position 50 instead of using the IUPAC code “Y,” representing the C/T polymorphic site. *PvDBP* sequences of all 37 Cambodian samples, Malagasy isolate *P*. *vivax* M15, and *P*. *vivax* Sal1 were aligned with ClustalW [26] based on default settings followed by manual editing in Sequence Alignment Editor v1.d1 [27]. Haplotype diversity was assessed using DnaSP v5.10.1 [28] with default settings. A phylogenetic tree was reconstructed using the maximum likelihood method implemented in RAxML [29] with 500 bootstrap replicates to assess clade support.

## Results

### A new type of *PvDBP* duplication is present in Cambodian isolates

**Fig 1:**
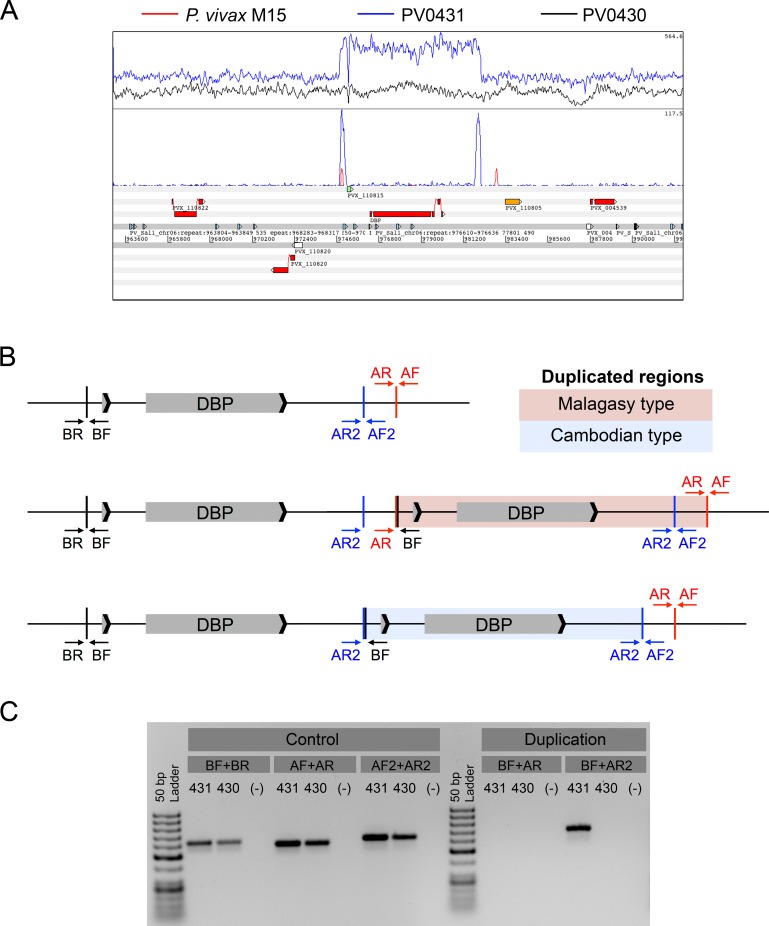
*PvDBP* duplication in Cambodian isolates. (A) Artemis genome view [21] showing whole-genome sequence coverage in the *PvDBP* region of PV0431 (blue trace) and PV0430 (black trace) for all reads (upper panel) and non-proper paired reads (middle panel). The Malagasy *P*. *vivax* M15 isolate’s non-proper paired reads (red trace, middle panel) suggest that its *PvDBP* duplication length is longer than that of PV0431. (B) Schematic of the *PvDBP* region and primer-binding locations in samples with a single *PvDBP* gene (top), a Malagasy *PvDBP* duplication region (middle), and a shorter Cambodian *PvDBP* duplication region (bottom). AR2+BF primers amplify both Malagasy and Cambodian breakpoints spanning the duplicated regions, while AR+BF primers only amplify Malagasy breakpoints. (C) Cambodian isolate PV0431 with a duplicated *PvDBP* region and PV0430 with a single *PvDBP* region, as determined by whole-genome sequencing. BF+AR primers failed to amplify the Malagasy *PvDBP* duplication in both Cambodian samples, while BF+AR2 primers successfully amplified the Cambodian *PvDBP* duplication in PV0431. AF2+AR2 primers detected *PvDBP* duplications in both Cambodian isolates, but not in DNA-negative controls (-).

Initial attempts to detect *PvDBP* duplications in Cambodian *P*. *vivax* samples using previously-published PCR primers [12] failed repeatedly (example shown in Fig 1C). To establish whether this was due to technical issues or the absence of *PvDBP* duplications in Cambodian *P*. *vivax* isolates, we used bwa [20] to map previously-generated Illumina sequence reads from individual Cambodian *P*. *vivax* isolates to the Sal1 reference genome [13]. Visual inspection of the alignments in Artemis [21] and LookSeq [22] clearly showed increased sequence coverage at the *PvDBP* locus in some isolates, including some where PCR-based detection had failed. Isolate PV0431 showed increased sequence coverage at the *PvDBP* gene region compared to flanking regions (Fig 1A, upper panel, blue trace), while isolate PV0430 showed even sequence coverage over these same regions (black trace). A *PvDBP* duplication was confirmed by identifying paired-end reads that mapped in the tail-to-tail, instead of the expected head-to-head, orientation (Fig 1A, middle panel, blue trace). To investigate whether this duplication was identical to the one previously identified in a Malagasy isolate [12], we mapped tail-to-tail reads for the Malagasy M15 isolate in the same manner (Fig 1A, middle panel, red trace). While the 5’ boundary of the duplication was identical in both genomes, the 3’ boundary was not, indicating that the tandemly-duplicated *PvDBP* region in Cambodian isolate PV0431 is shorter than that previously observed in Malagasy *P*. *vivax* isolates. Importantly, one of the previously-published PCR primers (AR) used to detect *PvDBP* duplication in Malagasy samples maps to the region that is not duplicated in these genomes, explaining how this primer failed to detect *PvDBP* duplication in Cambodian samples.

To investigate how widespread this new duplication is, we designed and tested new primers to amplify the alternative 3’ breakpoint observed in Cambodian isolate PV0431, hereafter referred to as the Cambodian, as opposed to the Malagasy, duplication (Fig 1B). We tested both new (AR2) and old (AR) 3’ primers on two Cambodian isolates that sequencing had identified as carrying (PV0431) or lacking (PV0430) a *PvDBP* duplication. As expected, the old BF+AR primers that detect the Malagasy duplication did not amplify a PCR product from PV0431, whereas the new BF+AR2 primers that detect the Cambodian duplication did amplify a PCR product of the expected size (Fig 1C). We detected no duplication in PV0430, which Illumina sequencing confirmed lacked a *PvDBP* duplication, and control primers confirmed that the regions flanking the *PvDBP* gene were present in both isolates. Importantly, the new BF+AR2 primers are able to amplify both Malagasy and Cambodian types of *PvDBP* duplication, but yield differentially-sized products.

These primers were then used to confirm the presence or absence of *PvDBP* duplication in all 35 whole-genome-sequenced Cambodian samples for which DNA was still available (Table 1). Identically-sized PCR products validated the Cambodian-type *PvDBP* duplication for 14/35 isolates tested, 11/11 of which contained both elevated coverage and the presence of tail-to-tail reads (no DNA remained for a 12^th^ sample with both elevated coverage and tail-to-tail reads). Duplication of *PvDBP* was supported in 2/3 additional isolates (PH0180-C, PH1113-C, PH1116-C) which lacked elevated coverage of *PvDBP* (1.17–1.38x) but did contain both tail-to-tail reads and intra-isolate SNPs. This suggests the presence of minor clones with the *PvDBP* duplication in these isolates, for which the limit of detection was reached in the case of PH0180-C. One additional isolate (PH0177-C), which lacked both tail-to-tail mates and elevated coverage *of PvDBP*, showed the *PvDBP* duplication by PCR. This may represent an additional minor clone not detected by whole-genome sequencing. Taken together, these results suggest that increased coverage, while least sensitive, is a useful marker for *PvDBP* duplications in this dataset. The presence of tail-to-tail read pairs and/or duplication-detecting PCRs, while not in complete agreement, have greater sensitivity and are likely both better able to detect *PvDBP* duplications in minor clones.

### Genetic diversity in *PvDBP* sequences

For the 12 isolates with *PvDBP* duplication supported by 1.8–2.8x coverage of *PvDBP* compared to average coverage of the entire genome, we reviewed intra-isolate SNPs in *PvDBP* to determine whether the two gene copies in a given isolate were identical. In 10 of the 12 isolates, there were no SNPs between the two *PvDBP* copies. In the other two isolates (PH1133-C, PH0182-C), there were SNPs at 30% and 70% frequency between the two *PvDBP* copies (Table 1), as well as SNPs at 30–70% frequency throughout the genome. These data suggest that these are polyclonal isolates, rather than clonal isolates containing two sequence-divergent *PvDBP* copies.

**Table 1:** Cambodian isolates with single and duplicated copies of *PvDBP*.

*PvDBP* status	WTSI ID	NIH ID	Average genome coverage	Average *PvDBP* coverage	*PvDBP* copy #	Tail-to-tail mate pairs (Y)	PCR supports *PvDBP* dup. (N/Y) †	Intra-isolate SNPs***
Single PvDBP*	PH0067-C		11.67	12.75	1.09		N	0
	PH0068-C		8.64	11.66	1.35		N	0
	PH0075-C		6.92	9.15	1.32		N	0
	PH0086-C		7.68	8.51	1.11		N	0
	PH0089-C		10.33	13.47	1.3		N	0
	PH0098-C		7.64	7.67	1		N	0
	PH0177-C		177.36	160.49	0.9		Y	0
	PH0180-C		57.69	70.6	1.22	Y	N	10
	PH0187-C		93.72	120.48	1.29		N	7
	PH0189-C	PV0430	113.45	123.58	1.09		N	0
	PH0310-C		71.63	74.04	1.03		N	0
	PH0320-C		94.67	79.45	0.84		N	0
	PH0385-C		35.84	43.12	1.2		N	0
	PH1078-C		13.94	13.72	0.98		na	0
	PH1109-C		50.66	50.44	1		N	0
	PH1111-C		5.3	6.67	1.26		N	0
	PH1112-C		17.18	19.25	1.12		N	0
	PH1113-C		18.58	21.75	1.17	Y	Y	2
	PH1116-C		11.98	16.52	1.38	Y	Y	15
	PH1117-C		5.78	6.58	1.14		N	0
	PH1121-C		10.29	12.16	1.18		N	0
	PH1124-C		18.06	17.8	0.99		N	0
	PH1126-C		10.9	9.95	0.91		N	6
	PH1131-C		7.25	8.87	1.22		N	0
	PH1132-C		6.26	6.82	1.09		N	0
Duplicated PvDBP**	PH0178-C		82.52	183.31	2.22	Y	Y	0
	PH0184-C		47.65	111.6	2.34	Y	Y	0
	PH0188-C		176.17	372.57	2.11	Y	Y	0
	PH0190-C	PV0431	152.9	427.71	2.8	Y	Y	0
	PH0309-C		21.37	49	2.29	Y	Y	0
	PH0182-C		25.68	46.37	1.81	Y	Y	28
	PH1084-C		6.89	15.06	2.19	Y	na	0
	PH1115-C		11.22	25.6	2.28	Y	Y	0
	PH1119-C		10.46	21.32	2.04	Y	Y	0
	PH1125-C		14.92	28.86	1.93	Y	Y	0
	PH1133-C		15.07	34.33	2.28	Y	Y	6
	PH1135-C		6.42	13.77	2.14	Y	Y	0

*Coverage of *PvDBP* 0.8–1.4x

**Coverage of *PvDBP* 1.8–2.8x

***Minimum of 5 reads supporting reference and alternative allele; allele frequencies 0.3–0.7

†All PCR results support only the shorter Cambodian *PvDBP* duplication

na, No remaining DNA available

**Fig 2:**
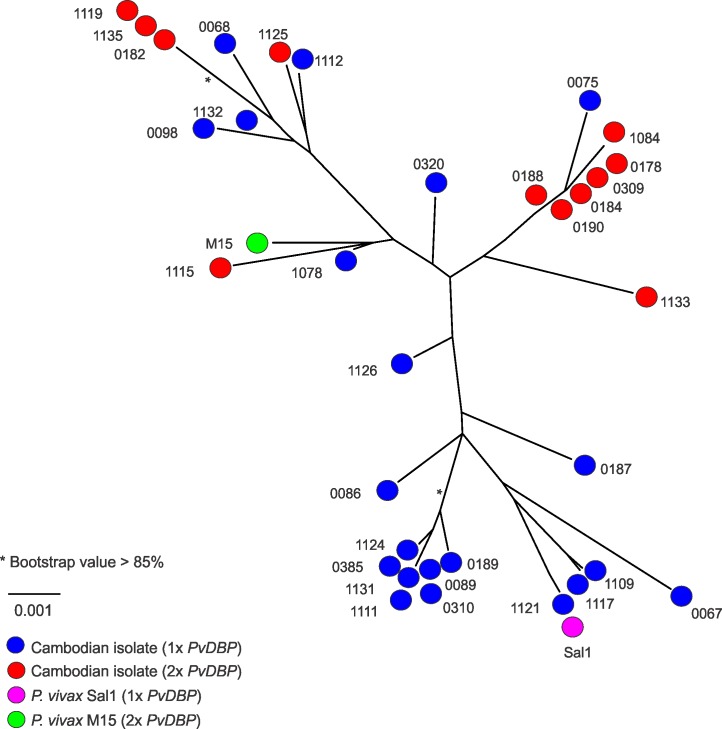
Maximum likelihood tree for *PvDBP* sequences in Cambodia. Maximum likelihood tree reconstructed based on alignment by ClustalW with bootstrap analyses to assess clade support (500 replicates) using a single consensus sequence for *PvDBP* (including introns) for 33 Cambodian isolates with *PvDBP* duplications (red) or single copies of *PvDBP* (blue) with numbers corresponding to the WTSI ID numbers in Table 1. Duplications were predicted by 1.8–2.8x average coverage in *PvDBP* compared to genome-wide average coverage, the presence of tail-to-tail mate pairs and PCR results (Table 1). *P*. *vivax* Sal1 containing one *PvDBP* gene (magenta) and Malagasy *P*. *vivax* M15 containing two *PvDBP* genes (green) are included as references. *bootstrap values >85%.

We aligned the *PvDBP* DNA sequences (including introns) of the reference *P*. *vivax* Sal1 line, Malagasy *P*. *vivax* M15 isolate, and all 37 whole-genome-sequenced Cambodian *P*. *vivax* isolates (including only the dominant haplotype when SNPs were present). Out of 3804 aligned bases, 51 sites were polymorphic (1.3%), excluding indels; we found unique *PvDBP* haplotypes in 77% (30/39) of samples, with a haplotype diversity of 0.98 ± 0.01. We categorized isolates as having either one or two *PvDBP* copies, excluding PH0177-C, PH0180-C, PH1113-C, and PH1116-C, which contained tail-to-tail read pairs and/or PCR support for duplication, but no increase in regional coverage. In a maximum likelihood tree analysis, isolates with either one or two *PvDBP* copies were intermixed and did not form distinct clades (Fig 2). This suggests that *PvDBP* duplication in Cambodian parasites is not the result of a single duplication event that has spread throughout the population. We found only three clades with bootstrap values >0.85, indicating that there are too few SNPs shared overall (likely due to outbreeding) to support the phylogenetic relationships between samples.

### Prevalence of *PvDBP* duplication in Cambodia, and its association with parasitemia and *DARC* genotype

**Fig 3:**
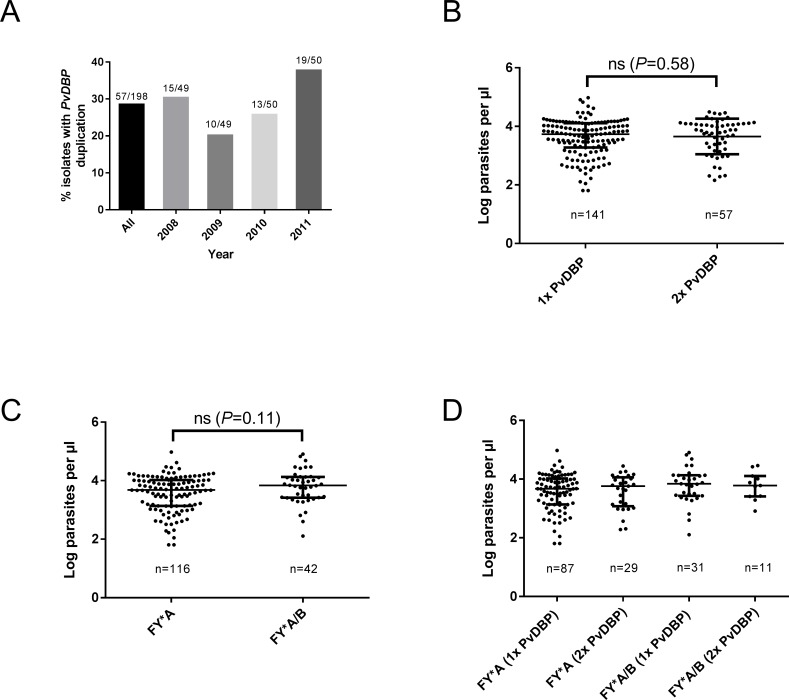
Prevalence of *PvDBP* duplication in Cambodia, and its association with parasite density and *DARC* genotypes. (A) The proportion of parasites carrying a *PvDBP* duplication (detected by PCR) over four years is shown. (B-D) *P*. *vivax* densities were not significantly different when stratified by (B) *PvDBP* copy number, (C) *DARC* genotype, or (D) combinations of *PvDBP* copy number and *DARC* genotype (Mann-Whitney test, *P>*0.05). Median (interquartile range, IQR) parasite densities are shown. ns, not significant. *P* values for 3D are reported in S1 Table.

We used the new BF+AR2 primers to measure the prevalence of *PvDBP* duplications in 198 Cambodian *P*. *vivax* isolates collected over four years in this seasonal transmission setting. None of these samples had been previously studied for *PvDBP* duplication by whole-genome sequencing or any other method. Overall, 29% (57/198) of *P*. *vivax* samples contained the duplication, with an annual prevalence ranging from 20% to 38% (Fig 3A). PCR product sizes suggested that all samples contained the Cambodian but not the Malagasy *PvDBP* duplication. This was confirmed by capillary sequencing of PCR products in 43 isolates with the duplication; in all cases, the duplication breakpoints occurred at an identical poly-T region marking the boundary of the Cambodian *PvDBP* duplication (linker sequence in S1 Fig). The 29% prevalence of *PvDBP* duplication in 198 Cambodian samples, as measured using the new BF+AR2 primers, is similar to the 32% (6/19) prevalence previously measured by whole-genome sequencing of a much smaller number of Cambodian isolates, although as noted this analysis only scored the presence of the duplication, not its precise boundary [13].

To assess whether *PvDBP* duplication is associated with higher parasite densities (which might suggest increased invasion efficiency) in Cambodian patients with malaria, we stratified parasite densities according to *PvDBP* copy number across these 198 newly-genotyped isolates. Parasite densities were not significantly different between parasites carrying one or two *PvDBP* genes (Fig 3B). There is no reported DARC-negativity in Cambodia, making it unlikely that the high prevalence of *PvDBP* duplication is associated with invasion into DARC-negative erythrocytes in this country. However, different *DARC* genotypes are present, and it has been recently found that susceptibility to *P*. *vivax* malaria episodes is higher in the FY*B/FY*B genotype relative to the FY*A/FY*A genotype, possibly due to significantly reduced binding of *PvDBP* to Fy(a+b-) erythrocytes [30,31]. To establish whether there is any association between FY*A or FY*B genotypes and *PvDBP* duplication in Cambodia, we sequenced the *DARC* genotypes of 159 patient samples that were also genotyped for *PvDBP* duplications. As shown in Fig 3C, 73% (116/159) of patients were FY*A homozygotes and 26% (42/159) were FY*A/*B heterozygotes. A single patient (1/159) was homozygous for FY*B. As expected, we found no evidence for the DARC-negative FY*B^*ES*^ allele in these 159 samples. Neither FY*A nor FY*A/*B were significantly associated with *PvDBP* duplications in this study population (Fig 3D).

### Global distribution of Cambodian and Malagasy *PvDBP* duplications

**Fig 4:**
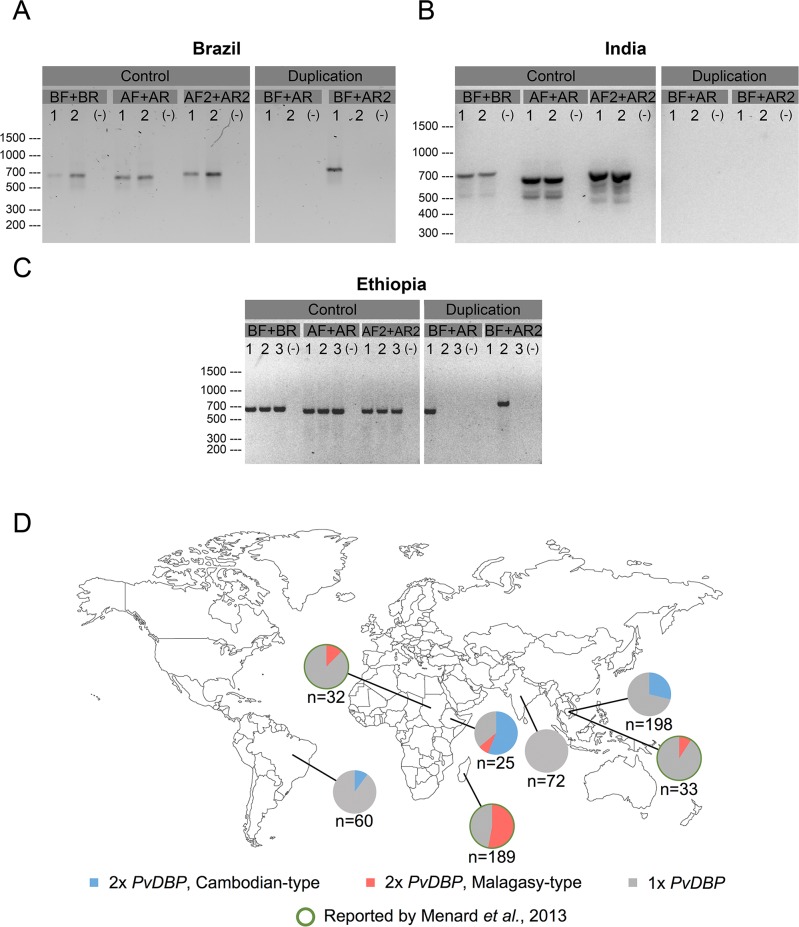
Global distribution of Cambodian and Malagasy *PvDBP* duplications. PCR primers that detect Cambodian and Malagasy *PvDBP* duplications were tested in three *P*. *vivax*-endemic countries (Brazil, India, Ethiopia). (A) Brazilian isolates 1 and 2 contain a Cambodian *PvDBP* duplication and a single *PvDBP* region, respectively. Note that the sizes (and sequences, S1 Fig) of the PCR products match the Cambodian *PvDBP* duplication detected by the BF+AR2 primers, but not the Malagasy *PvDBP* duplication detected by the BF+AR primers. (B) Indian isolates 1 and 2 lack Cambodian and Malagasy *PvDBP* duplications. (C) Ethiopian isolates 1, 2, and 3 contain a Malagasy *PvDBP* duplication, a Cambodian *PvDBP* duplication, and a single *PvDBP* region, respectively. (A-C) All isolates show bands for the positive control AF2+AR2 primers, and no bands for DNA-negative controls (-). (D) In the present study, the prevalence of Cambodian *PvDBP* duplication is 0/72 in India, 6/60 in Brazil, 14/25 in Ethiopia, and 57/198 in Cambodia, and the prevalence of Malagasy *PvDBP* duplication is 0/72 in India, 0/60 in Brazil, 2/25 in Ethiopia, and 0/198 in Cambodia. In a previous study [12], the prevalence of Malagasy *PvDBP* duplication was 4/32 in Sudan, 100/189 in Madagascar, and 3/33 in Cambodia.

To determine whether the Cambodian *PvDBP* duplication is present outside Cambodia, we used the new BF+AR2 primers to amplify the *PvDBP* region in *P*. *vivax* clinical isolates, none of which had been previously analysed for *PvDBP* duplication, from three different continents. While we did not detect Cambodian or Malagasy *PvDBP* duplications in 72 samples from Goa, India (Fig 4B), we found Cambodian *PvDBP* duplications in 10% (6/60) of samples from Brazil (Fig 4A) and 56% (14/25) of samples from Ethiopia (Fig 4C). Interestingly, Malagasy duplications were also found in samples from Ethiopia (Fig 4C) but not Brazil (Fig 4A). These data, combined with those previously published for the Malagasy *PvDBP* duplication [12], indicate that both types of *PvDBP* duplication are globally distributed (Fig 4D).

## Discussion

Although *P*. *vivax* causes the majority of malaria cases in Asia, South America, and the Pacific, research of the *P*. *vivax* genome lags significantly behind that of the *P*. *falciparum* genome. While thousands of *P*. *falciparum* clinical isolates have already been sequenced and analyzed, revealing insights into global epidemiology, as well as the origin and spread of drug resistance [32–34], only a handful of *P*. *vivax* genome sequences were publicly available until recently. Genomic data from four isolates suggested that *P*. *vivax* diversity may be more extensive than *P*. *falciparum* diversity [35], and sequencing additional isolates from Madagascar and Cambodia revealed CNVs and indels in genes associated with erythrocyte invasion [12,36], including a *PvDBP* gene duplication. Given the number of samples in these studies, however, it was not possible to infer whether any of these features are confined to individual infections or geographic locations, or are much more widespread and common forms of variation. The recent publication of more than 200 *P*. *vivax* genomes from across the world revealed that CNV at the *PvDBP* locus is one of the most common CNVs in the *P*. *vivax* genome [13]. In contrast, no *PvDBP* duplications were found in a concurrent population genomics study of 182 *P*. *vivax* isolates from 11 endemic countries [37]. This discrepancy may be due in large part to the reliance on the Malagasy duplication primers for validation in the latter study, which as noted here will miss the newly-identified Cambodian duplication [37]. In this study, we used sequence data from clinical isolates [13] to infer that *PvDBP* duplications in Cambodia had different boundaries than the *PvDBP* duplication in Madagascar [12]. Using these sequence data to define new duplication-specific PCR primers, we defined the boundaries of this new duplication type, showed that it was present at a prevalence between 20% and 38% over four consecutive transmission seasons in Cambodia, and that it was also present in *P*. *vivax* infections in Ethiopia and Brazil, although absent from a study site in India. In Cambodia, there was no association between *PvDBP* duplication and either parasite burden or *DARC* genotype. Thus, there are at least two common forms of *PvDBP* duplication with global distribution; the duplication-specific PCR primers defined in this study will help to estimate their prevalence in other *P*. *vivax*-endemic regions and enable genotype-phenotype correlation studies.

One key question is whether the Cambodian and Malagasy duplications arose independently in specific locations and then spread around the world, or whether *PvDBP* CNVs are continuously arising *de novo*, but consistently occur at the same defined boundaries, leading to either “Malagasy-type” or “Cambodia-type” *PvDBP* duplications. Phylogenetic analysis at this stage favors the latter hypothesis. *PvDBP* is known to be under positive selection pressure [38], with widespread diversity even within the DARC-binding region, Domain II. Of the 12 Cambodian isolates that have been whole-genome sequenced and have *PvDBP* duplications, ten have duplicated *PvDBP* genes that are identical in sequence. In phylogenetic trees, these sequences are interspersed with *PvDBP* sequences from samples without duplications, strongly suggesting that the Cambodian duplication arose independently multiple times on different genetic backgrounds. An alternative explanation is that the Cambodian duplication arose only once, and the two *PvDBP* genes subsequently diverged due to immune selection pressure. Under this hypothesis, frequent gene conversion would be required to maintain sequence identity between the two *PvDBP* copies within a given isolate. Whether this is likely is unclear, as little is known about the frequency of gene conversion in *Plasmodium* parasites, although this phenomenon of shared mutations between identical adjacent regions of the genome has previously been seen in other invasion genes in *P*. *falciparum* [39]. At this moment, however, independent origins of *PvDBP* duplication at defined amplification hotspots appears to be the simplest explanation.

The nature of the *PvDBP* duplication boundaries also favors this hypothesis and hints at a potential molecular mechanism. While there is no homology between the 3’ boundaries of the Cambodian and Malagasy duplications, both occur in homopolymeric T tracks, which are not as frequent in the *P*. *vivax* genome as they are in the AT-rich *P*. *falciparum* genome. Recent *in vitro* studies selecting for *P*. *falciparum* parasites with resistance to DSM1, an inhibitor of dihydroorotate dehydrogenase (DHODH), produced multiple lines with CNVs at the *PfDHODH* locus [40]. These CNVs had different boundaries, but notably all of them contained homopolymeric A or T tracks, suggesting that such tracks facilitate frequent localized recombination and CNV generation during DNA replication. Such CNVs may provide raw material for evolution, by providing a second copy of a given gene or enabling the accumulation of mutations in one copy while limiting the potential risk of deleterious mutations by maintaining a second wild-type copy. In *Plasmodium* parasites, which are faced with numerous strong selection pressures in both humans and mosquitoes, the advantages of such a mechanism are obvious. While it is not known whether this mechanism is common, the parallels between *PfDHODH* and *PvDBP* CNVs are intriguing, and a genome-wide analysis of common CNVs could be revealing.

What then is driving CNVs at the *PvDBP* locus? The best known examples of CNVs at *Plasmodium* genes involve antimalarial drug resistance; for example, selection pressure from mefloquine amplifies the *MDR1* gene in *P*. *falciparum* [41] and perhaps also in *P*. *vivax* [13]. Antimalarial drug pressure appears unlikely in this context, although not inconceivable; for example, if *PvDBP* duplication confers an increased growth rate to *P*. *vivax* parasites, this could provide an advantage against some slow-acting drugs. However, given the critical role of the PvDBP-DARC interaction in *P*. *vivax* erythrocyte invasion, it is likely that CNVs at *PvDBP* are being selected by genetic variation in *DARC*. Is this variation DARC-negativity? The presence of *PvDBP* duplications at frequencies approaching 40% both globally [12,13], and over multiple transmission seasons in Cambodia where DARC-negativity is essentially absent (Fig 3), makes this unlikely. Another possibility is that *PvDBP* duplications increase the efficiency of parasite invasion into erythrocytes carrying other common *DARC* genotypes, such as FY*A or FY*B. No association between *PvDBP* duplication and these *FY* genotypes was seen in this study, but the fact that *P*. *vivax* clinical isolates were obtained only from patients with acute malaria episodes may confound this analysis. A much larger study that genotypes both the *DARC* and *PvDBP* loci, is conducted where the prevalence of DARC-negativity is high, and includes both asymptomatic and clinical cases is needed to comprehensively test for association between *PvDBP* duplication and *DARC* genotype. In addition, testing the functional consequences of *PvDBP* duplication, through protein binding assays or *ex vivo* growth assays, are clearly needed. Since *Plasmodium* parasites are highly adaptable, understanding how they respond to human erythrocyte variation will be particularly relevant to studies of naturally-acquired immunity to PvDBP and the development of any PvDBP-based vaccine.

## Supporting Information

S1 Checklist STROBE Checklist of items that should be included in reports of cohort studies. (DOC)

S1 Fig Cambodian *PvDBP* duplication occurs at identical 5’ and 3’ poly-T regions. Fasta file representing the 736-bp PCR product linking the duplicated regions in samples with the Cambodian-type duplication. Primer sites for BF (red), AR2 (blue), and the poly-T break point (bold black) are shown.
(TIF)

S1 Table *P* values from Mann-Whitney tests comparing association of *PvDBP* copy number and *DARC* genotypes with parasite density displayed in Fig 3D. (XLSX)
